# The landscape of nonlinear structural dynamics: an introduction

**DOI:** 10.1098/rsta.2014.0400

**Published:** 2015-09-28

**Authors:** T. Butlin, J. Woodhouse, A. R. Champneys

**Affiliations:** 1 Department of Engineering, University of Cambridge, Trumpington Street, Cambridge CB2 1PZ, UK; 2 Department of Engineering Mathematics, University of Bristol, Queen’s Building, Bristol BS8 1TR, UK

**Keywords:** nonlinear structural dynamics, mind-map, ontology, vehicle brake squeal, cable-stayed bridges

## Abstract

Nonlinear behaviour is ever-present in vibrations and other dynamical motions of engineering structures. Manifestations of nonlinearity include amplitude-dependent natural frequencies, buzz, squeak and rattle, self-excited oscillation and non-repeatability. This article primarily serves as an extended introduction to a theme issue in which such nonlinear phenomena are highlighted through diverse case studies. More ambitiously though, there is another goal. Both the engineering context and the mathematical techniques that can be used to identify, analyse, control or exploit these phenomena in practice are placed in the context of a mind-map, which has been created through expert elicitation. This map, which is available in software through the electronic supplementary material, attempts to provide a practitioner’s guide to what hitherto might seem like a vast and complex research landscape.

## 1. Introduction

While there have been several decades of developments of mathematical models and analysis techniques of nonlinear dynamical phenomena, as yet these have had relatively little impact in practical structural engineering. One of the reasons for this is that structural dynamics often limits itself to small-amplitude vibrations, with a fundamental paradigm being to keep the amplitude of vibration as low as possible. In this limit, it is fair to say that linear effects often dominate. Another reason is that the frequency domain is an extremely useful paradigm to analyse linear dynamics in engineering structures with many degrees of freedom, yet nonlinear effects are most readily observed and analysed using time-domain models with only a few degrees of freedom. So there is often a mismatch between engineering practice and mathematical theory.

While linear techniques have often sufficed in the past, there is an increasing need to take nonlinear effects into account in modern engineering design. This can be seen as we push the envelope of engineering design into: more flexible regimes, where larger-amplitude motions are likely to occur; higher speeds, where nonlinear fluid–structure interaction is significant; and tighter tolerances, where understanding contact and friction dynamics becomes especially important. This last category is a particular challenge because of the fundamental failure of linear methods to deal with harsh or non-smooth effects, such as stick–slip friction and vibro-impact. These topics remain an area of active research where theoretical developments need to go hand in hand with carefully calibrated experiments and practical solutions.

Of course, nonlinear dynamical effects occur in many areas of science and mathematics. This theme issue on ‘A field guide to nonlinearity in structural dynamics’ is associated with one particular area of nonlinear science, albeit one with important academic and industrial applications. Moreover, structural engineering perhaps has not attracted as much attention as applications in, for example, bioengineering, neuroscience, fluid dynamics or weather forecasting. However, as will be seen, our chosen theme covers topics as diverse as aircraft and helicopter dynamics, micro-electro-mechanical systems (MEMS) and the motion of a bowed violin string.

The core aim of the collaboration is to exploit nonlinear effects in order to improve the dynamic performance of engineering structures to solve practical problems while at the same time advancing the theoretical underpinnings of the subject. The collaborators come from very diverse backgrounds, and it quickly became apparent that the subject landscape itself is complicated enough that experts within the broad field of ‘nonlinear structural dynamics’ do not all speak the same language. This article presents an attempt to map the landscape of nonlinear structural dynamics: the map is intended to represent and connect the islands inhabited by different specialists. With this map as an over-arching guide, the theme issue then contains technical papers selected to touch on a significant portion of the landscape from both industrial and academic perspectives.

The map began with a knowledge-capture exercise within a collaborative group that had come together in the UK through a national-level consortium funded by the Engineering and Physical Sciences Research Council: theoretical and experimental methods, application areas, concepts and jargon habitually referred to were collected. The open-source software ‘designVue’ (http://www3.imperial.ac.uk/designengineering/tools/designvue) was then used to organize these into a structured ‘mind-map’, which after several iterations now takes the form of a hierarchical structure of labels intended to capture observations, concepts and modelling tools within the field of nonlinear structural dynamics. It could be viewed as the beginning of an ‘ontology of nonlinear structural dynamics’. It is not intended to be a finished product: on the contrary, the current version of the map can be found in various forms in the electronic supplementary material, and the authors actively invite readers to re-use the material and to provide personal feedback.

Two general comments should be made at the outset. First, any individual reader is likely to see in the map things they are familiar with, and other things which may make them ask ‘Why on earth is that there?’. This is not necessarily a fault in the map, but a reflection of the intention to represent many different perspectives on the general subject. Second, there are of necessity many areas of the wider field of nonlinear dynamics that are not considered here, both mathematical studies and application areas remote from the specific field of structural dynamics. This omission does not reflect any view by the authors that these missing subjects are less important: if a reader from one of these omitted areas were to be inspired to extend the map to encompass their interests, then the authors would be delighted.

## 2. Overview of a complex landscape

For a mind-map to be effective, it should be useful when viewed from a number of different contexts and perspectives. One over-arching perspective kept in mind while compiling the map has been that the map should provide a ‘consultant’s checklist’. Faced with an apparently nonlinear vibration phenomenon in a new context, how should one begin to understand and explore it? There are many questions one might ask, and many possible avenues for progress, but no single expert is likely to sympathize with or remember them all. Some of the necessary questions express scepticism: ‘Are you really sure about …?’ Others probe specific features that may or may not be present in the particular application: these can relate to the measurement and interpretation of empirical findings, or to theoretical concepts, such as ‘Do you see sudden jumps in vibration regime when a parameter is varied continuously?’ Yet others relate to expectations and desired outcomes: ‘Were you expecting nonlinear effects in this problem?’ ‘Is the nonlinear phenomenon something you want to eliminate, or to encourage?’ Exploring such questions can lead towards an understanding of available techniques, precedents in other applications, what kind of experts need to be consulted, and what kind of outcomes might be expected from different styles of theoretical modelling or experimental investigation.

This starting point should not preclude other perspectives. For example, someone entering the landscape from a particular application- or technique-specific direction might seek to find connections to other related nonlinear problems in order to gain insight and new solutions, or they may wish to find connections to other theoretical perspectives or mathematical techniques. It is also hoped that the map might provide a way of generating new ideas, new research questions, new solution methodologies or, ultimately, new designs that exploit or ameliorate nonlinear phenomena to commercial or societal advantage.

**Figure 1.**
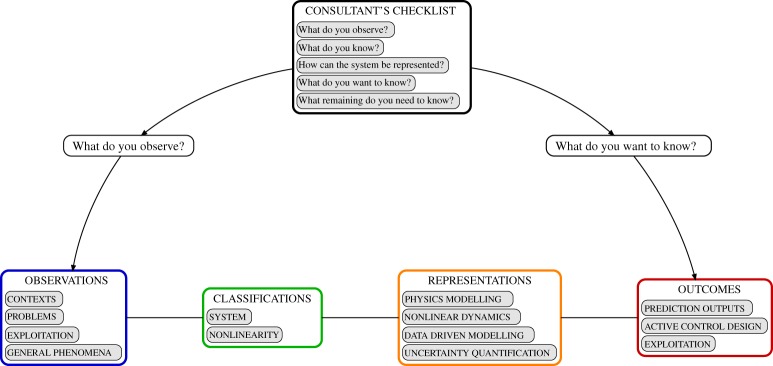
Overview of the proposed structure of a consultant’s checklist, or ‘mind-map’, of nonlinear structural dynamics (relabelled snapshot from designVue).

Nevertheless, one has to start somewhere and figure 1 shows an outline of the structure of the map when viewed from the perspective of a consultant’s checklist. There are two fundamental questions that can be asked of any structural vibration problem: ‘What do you observe?’ and ‘What do you want to know?’ These provide a starting point for categorizing nonlinear structural dynamics from two points of view: *observations* (far left, colour-coded blue) and *outcomes* (far right, colour-coded red). The *observations* group includes specific problems (e.g. brake noise) or more general phenomena (e.g. friction-induced vibration). Not all nonlinear effects are undesirable, so both problematic phenomena and exploitations of nonlinearity are included in this group. The *outcomes* group contains properties of the system behaviour that the user would like to be able to predict either qualitatively or quantitatively, as a step towards a higher-level goal such as design optimization, or for providing insight into the essence of the phenomenon.

These two groups form the usual starting point in an industrial context, where commercial drivers demand a practical point of view. However, it often contrasts with the typical starting point in academic work, encapsulated by the intermediate categories of the map. There are two new groups. First comes system *representations* (mid-right, colour-coded orange): ways in which the underlying system dynamics can be modelled or characterized and which may have predictive capabilities that give some of the desired *outcomes*. Different methods will yield different kinds of predicted quantities. Looking in the other direction, there is a very wide range of methods for representing observed phenomena, but they are not, of course, equally applicable to all phenomena. This leads analysts to discuss higher-level abstract features or *classifications* (mid-left, colour-coded green) of nonlinear structural dynamics problems. A given representation may be particularly well matched to a set of classifications, and where this pattern also characterizes a phenomenon, the combination may indicate suitability of a method for a given phenomenon. This type of ‘mapping’ is currently carried out intuitively by experts, drawing on their own experience. By creating a more structured map there is potential for identifying unexpected links between subjects, or at least for ensuring that important things are not overlooked.

Each of these top-level groups (*observations*, *classifications*, *representations* and *outcomes*) includes a subsequent hierarchy of sub-labels or nodes. The full map can be found in the electronic supplementary material as a designVue document. At the time of writing, it contains 302 nodes (not including subheadings) that span the subject domain. Digging into these details is best done through a case study: two examples are presented in §§4 and 5.

The map structure naturally lends itself to a systematic study of the connectivity between nodes in adjacent groups. But there are challenges to developing such a study; first in choosing and labelling nodes in an unambiguous and ‘complete’ manner, and second in populating a description of node connectivity. Such studies, often labelled as ‘ontologies’, are an active area of research (e.g. [1–3]), and these challenges are common to all such work. The map presented here is a pragmatic first step, and the goals are to provide a tool that:

— is available for use even during its development;
— can be readily visualized in a variety of ways;
— can usefully be navigated using open-source software; and
— is itself open-source and can be customized and tailored for specific applications.

The map can be used in cases where there is incomplete knowledge, by initiating a systematic way to guide an industrialist towards identifying: (a) the current state of knowledge of their problem or phenomenon to exploit; (b) gaps in this knowledge; (c) system representations that might be most appropriate; and (d) relevant experts who may be able to help. Starting from the two questions ‘What do you observe?’ and ‘What do you want to know?’ and simply working through the map does not necessarily yield insight, however, so the following is a suggested set of questions designed to identify what is really known, and perhaps to identify conflicts with what is currently assumed.

(1) What do you observe?
  • What is the evidence for nonlinearity in the system behaviour?
    ∘ Which tests have been performed to identify nonlinearity in the response? For example:
      — vibration is self-sustained (nonlinearity must be involved);
      — narrow-band input: generation of harmonics (nonlinearity is present, but care is needed to check that this is not an artefact of an experimental set-up);
      — amplitude dependence of response (does the response scale non-trivially with changing excitation amplitude?);
      — jumps in behaviour when a parameter is continuously varied (is there a step change in behaviour however slowly the parameter is varied?);
      — sensitive dependence on initial conditions (is it difficult to produce repeatable results?);
      — poor coherence (do repeated transfer function measurements give poor coherence? This is not a robust check, as there are reasons other than nonlinearity that can cause this, and good coherence does not necessarily prove that nonlinearity is absent);
      — bistability (can two states exist under the same conditions?); and
      — sweep up/sweep down (is the behaviour independent of sweep direction for sinusoidal sweep tests?).
    ∘ Which ‘general phenomena’ are observed?
  • What are the candidate mechanisms that can lead to this phenomenon? What are the other possibilities?
  • How would the phenomenon be described without jargon?
  • Can what is actually known about the phenomenon be separated from what is hypothesized or assumed?
  • Have all experimental nonlinearities been eliminated or characterized (e.g. electromagnetic shakers with friction and maximum throw; boundary effects such as friction damping from clamping; sensor nonlinearity)?
  • Are high-amplitude regimes triggered or do they evolve smoothly?
  • Have all the input excitation sources been identified and to what extent have they been characterized? Could they be the source of the phenomenon rather than intrinsic nonlinearity?
  • To what extent can experimental test conditions mimic operational conditions? In other words, are test conditions representative? Would nonlinear effects be more or less likely to be important during normal operation?
  • Can the phenomenon be reproduced in a controlled test?
  • What is the simplest experiment that can reproduce the phenomenon?
(2) What do you know?
  • What is the pattern of categories that best match the problem?
  • Which subheadings are beyond current knowledge?
  • What would be required to fill that gap? More tests/speaking to an expert?
(3) How can the system be represented?
  • What are the simplest models that can reproduce the phenomenon? Sometimes more than one underlying critical model ingredient can give rise to apparently similar phenomena (e.g. negative damping/mode coupling in brake squeal).
  • It is common to compute ratios of fast Fourier transforms of input and output signals: but for nonlinear systems a great deal of care is needed, as their meaning is not obvious and the results will depend on the excitation method, input amplitudes and other factors.
  • Which aspects of the problem are beyond current characterization?
  • What would be required to fill that gap? More tests/speaking to an expert?
(4) What do you want to know?
  • What is the urgency of the problem: is this a long-term research problem or is a short-term fix required?
  • What is the end-goal of investigating the phenomenon? Elimination, exploitation, optimization, operating condition avoidance or characterization?
  • What is the problem severity? Safety critical, performance critical or a nuisance?
  • What is of most interest to be able to predict with a system representation? What are the metrics of interest? Or is qualitative behaviour, and an understanding of the underlying effects of key model ingredients, more important?
  • What does the problem currently prevent in terms of capability? Or what would be possible if the phenomenon could be fully exploited?

Each of these questions is intended to produce a list of answers, where the mind-map can serve as a library of suggestions. For particular sub-domains within the field of nonlinear structural dynamics, it may be feasible to lay out a specific ‘handbook’ where a standard set of suggested procedures can be followed: the final contribution to this theme issue is a procedural guide for the sub-domain of *localized*^(2.2.1.1)^, *weak*^(2.2.3.3.1)^ nonlinearity [4] (the superscripts correspond to the reference numbers of these items in the map). But currently this is infeasible for the whole field, as methods are still under active research, and there remain many open questions about the best ways to approach different problems. The approach taken here is deliberately open-ended and is intended to be useful for generating unexpected leads and helping a user to see beyond their own perspective.

Although the initial motivation behind the development of the map was for a ‘consultant’s checklist’, it is apparent that the map provides a structure to which various agendas can be brought. It is as equally applicable to academia as to industry, and could be used in both contexts as a networking guide, a lateral-thinking aid or a tool for identifying knowledge gaps.

## 3. Theme issue contributions

The contributions selected for this theme issue provide a sampling of research from the field of nonlinear structural dynamics, covering recent advances in a wide range of topics: nonlinearity localization [4], friction modelling [5], nonlinear damping [6], nonlinear statistical energy analysis [7], normal forms [8], Bayesian system identification [9], bifurcation analysis of industrial structures [10], nonlinear MEMS oscillator design [11], machine tool vibration [12] and helicopter rotor stability [13].

**Figure 2.**
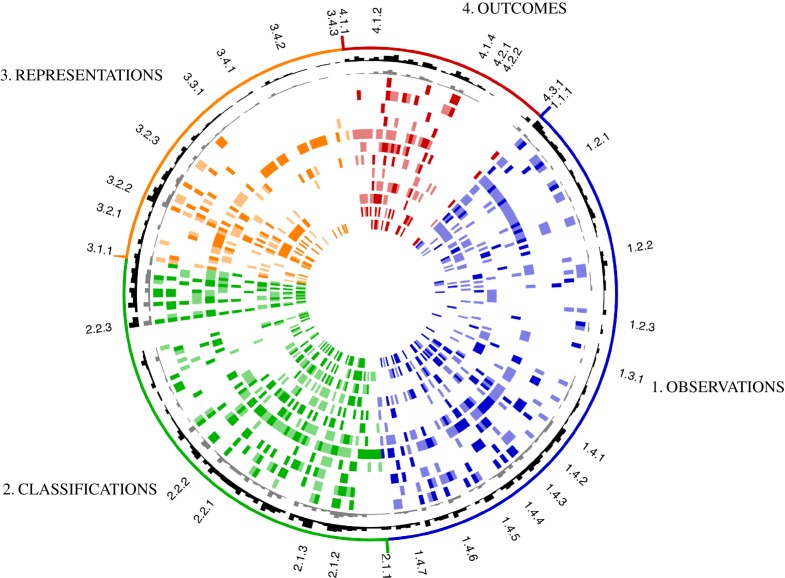
Summary of how each contribution fits within the context of the map. Each label within the map corresponds to an angular position (labels available in the electronic supplementary material). Colour-coding corresponds to *observations* (blue), *classifications* (green), *representations* (orange) and *outcomes* (red). Dark and light shades correspond to ‘direct’ and ‘indirect’ relevance, respectively. The two innermost circles represent the case studies in §§4 and 5, and the contributing articles are arranged radially outwards according to their order cited within this article [4–13].

These contributions were not selected with specific reference to the mind-map, but the map gives a good way to summarize the coverage of the various articles and also to show how much of the landscape of the subject is captured here. Each author has identified how their paper fits within the context of the map by filling in a questionnaire in which they rated all 302 labels in the current map as directly relevant, indirectly relevant or not relevant to their contribution. Figure 2 shows a visual overview of the result. Node labels have been removed in this version, but the colour-coding of the boxes in figure 2 has been retained and the heading structure (to three levels) is shown for reference: this figure is simply intended to illustrate the way in which the contributions span the map. Full details are available in the electronic supplementary material.

Each contribution is represented by a ring-shaped chart. The two innermost rings refer to the case studies treated in some detail in §§4 and 5, while the remaining rings represent the contributed articles, arranged radially outwards according to their order cited within this article [4–13]. The colour intensity denotes ‘indirect’ (pale) or ‘direct’ (dark) relevance. The two outer rings indicate the sum along each radial line: the outermost ring shows the total score, the penultimate one includes only ‘direct’ relevance. The details of how each contribution links to the full map can be found in the electronic supplementary material.

Using this classification, the contributions are related directly to 74% of the map and indirectly to 92% of the map. To some extent, this high coverage is self-fulfilling as the author contributions are not wholly independent of the map generation. Nevertheless, it illustrates the wide range of topics spanned by the contributions in this theme issue and provides a rational structure for: (a) identifying how contributions may relate to each other; (b) identifying how other studies fit within the overall landscape of nonlinear structural dynamics; and (c) growing the map to include new items. Some of the patterns revealed in this figure will be discussed in §6.

It is neither practicable nor of great interest to describe in words every detail of the map: instead, two case studies are used to show how it can be applied to specific phenomena. To avoid repeating material covered in other articles in this theme issue, topics have been chosen that are not covered in detail elsewhere: vehicle brake squeal and cable-stayed bridge dynamics. A detailed discussion of vehicle brake squeal follows, followed by a more brief presentation of cable-stayed bridge dynamics to illustrate a few corners of the map that are not covered by brake squeal.

## 4. Case study I: vehicle brake squeal

Brake squeal is an undesirable phenomenon that causes trouble and expense for manufacturers in the automotive sector. When a vehicle brake is applied, there is obviously a sliding frictional contact between the brake liners and the disc or wheel rim (depending on the type of brake). Sometimes an instability occurs so that, instead of steady sliding at this interface, vibration is generated, which in turn leads to sound radiation. The resulting noise can be described by a variety of terms such as ‘groan’ or ‘judder’, but the generic term ‘squeal’ is used here to cover all such effects, regardless of frequency range. Vehicle and component manufacturers would very much like to understand squeal well enough that they could reliably design non-squealing brakes.

It will be seen that the map provides a structured checklist for approaching a nonlinear structural dynamics problem like this (or indeed a nonlinear phenomenon for exploitation), and helps give confidence that key points have not been missed. The convention used in the following discussion will be to italicize text that corresponds to labels within the map, together with a superscript reference number that allows identification of its place within the heading structure (see the electronic supplementary material).

Using the questions listed in §2 and the map as a guide, a picture of the phenomenon of vehicle *brake squeal*^(1.2.2.1)^ emerges. It will be seen that the common themes highlight the connections between vehicle *brake squeal* and several other fields: bowed string *musical instruments*^(1.1.3)^, which rely on *friction-induced vibration*^(1.4.5.7)^ to generate sound [14], *machining*^(1.1.7)^, where some forms of cutting instability are related to squeal [15], and *stick–slip*^(1.4.5.8)^ torsional oscillation during *oilwell drilling*^(1.1.9)^, where the nonlinear *self-sustained oscillation*^(1.4.5.2)^ that develops closely resembles some forms of squeal [16,17].

The details of the connections between vehicle *brake squeal* and the rest of the map can be found in the electronic supplementary material. The links have been generated to the best of the authors’ knowledge and are a subjective opinion: other experts in this field may come to different conclusions, but it is anticipated that such differences would not alter the overall picture very much. A discussion of these links follows that, for the sake of brevity and interest, is not exhaustive: only links of particular interest are highlighted.

### (a) ‘What do you observe?’

This question yields some very clear answers in the context of vehicle *brake squeal*. The evidence for nonlinearity in this case is obvious, as it is a classic example of *self-sustained*^(1.4.5.2)^ vibration, and *self-sustained* vibration must involve nonlinearity. *Friction*^(3.1.1)^ plays a key role in squeal through one mechanism or another, so it is not surprising that other similar observed phenomena include *squeak*^(1.2.2.3)^, which differs from steady-sliding squeal as the underlying sliding contact repeatedly reverses sign [18], and *bowed string instruments*^(1.3.1.1)^, as discussed above. Bowed string instruments differ from squeal in an important way: in this case, *self-sustained* vibration is desirable and necessary for producing sustained notes. This fundamentally changes the way in which the two problems are approached, as the desired *outcomes*^(4)^ are opposite: for squeal the primary concern is to be able to predict its onset or *bifurcation threshold*^(4.1.3.3)^ (and perhaps *amplitude*^(4.1.3.4)^ and *frequency*^(4.1.3.2)^), whereas for *bowed strings* the *detailed motion*^(4.1.3.9)^ of the *transient*^(4.1.3.16)^ and *steady-state response*^(4.1.3.17)^ are of great interest in order to *optimize*^(4.3.1)^ instrument design. Another example of the *exploitation*^(1.3)^ of *friction-induced vibration*^(1.4.5.7)^ is for *ultrasonic motors*^(1.3.8)^: in this context vibration is used to drive steady motion through a frictional contact [19]. Some models of squeal have a lot in common with models of *wing flutter*^(1.2.2.4)^: the main difference being that the underlying physics is quite well understood for flutter, but the physics of friction, needed for squeal prediction, is still an active research topic [5].

On a conceptual level, vehicle *brake squeal*^(1.2.2.1)^ exhibits more general phenomena associated with nonlinear systems, such as *mode locking*^(1.4.1.3)^, *friction and joint damping*^(1.4.2.2/3)^, *mode transitions*^(1.4.3.1)^, *spatio-temporal pattern formation*^(1.4.3.5)^, *hysteresis*^(1.4.6.1)^ and *sensitivity to initial conditions or test conditions*^(1.4.6.9)^. The term *mode locking* is a good example of an item in the map where different experts use similar jargon with very different meanings: *mode locking* can refer to systems involving the coupling of more than one underlying linear mode (e.g. [20]); or to phenomena arising from coupling two systems that have closely matched resonant frequencies (e.g. [21]). The latter sense is more relevant to squeal: it has been shown that this kind of mode ‘matching’, ‘coupling’ or ‘locking’ can sometimes play a significant role, although it is by no means the whole story. The other general nonlinear effects associated with squeal are somewhat better defined. *Friction and joint damping* are ever-present in assembled structures and, although it is not the cause of *brake squeal*, it is known that damping plays a crucial role [22]; *mode transitions* have been observed when squeal frequencies switch unexpectedly between and also within experimental tests; a rich variety of *spatio-temporal pattern formation* phenomena have been observed and simulated, which is an area of research coming under increasing attention [23]. Another feature often associated with self-sustained oscillation is that of *hysteresis*, where the operating conditions that lead to the initiation of vibration are different from those where it is no longer sustained, e.g. the normal pre-load required to trigger squeal is often notably higher than the pre-load at which squeal stops [24]. Finally, one of the most notorious features of squeal is its *sensitivity to initial conditions or test conditions*, which makes it such a difficult (and interesting) phenomenon to study. Such sensitive dependence is a very familiar phenomenon in the wider field of nonlinear systems theory (e.g. the ‘chaos butterfly’ [25]).

Examples of self-sustained vibration are rather amenable to experimental testing, as problems due to input excitations become irrelevant (e.g. nonlinearity associated with electromagnetic shakers). On the other hand, the phenomenon is often associated with poor reproducibility, because the details depend so much on the underlying nonlinearity that sustains the vibration: for the case of *brake squeal*, and also for other examples of friction-driven vibration, the nonlinearity is not generally very well characterized. Finally, the question ‘What is the simplest experiment that can reproduce the problem?’ raises some challenges. It is not difficult to create an experimental test rig that squeals (that is to say, exhibits *friction-induced*^(1.4.5.7)^ *self-sustained vibration*^(1.4.5.2)^); probably the simplest case is that of a spring-restrained *mass resting on a moving belt*^(3.2.1.5)^. However, the extent to which this mimics operating conditions of a disc brake is questionable, as the time scales, geometry and number of relevant modes all differ substantially. This is a challenge relevant to all experimentalists: that of designing an experimental test rig that simplifies the phenomenon enough to create a tractable problem (one that can be instrumented and meaningfully correlated to a *system representation*^(3)^) yet retains the key features that make the actual phenomenon under operating conditions difficult to predict.

### (b) ‘What do you know?’

**Figure 3.**
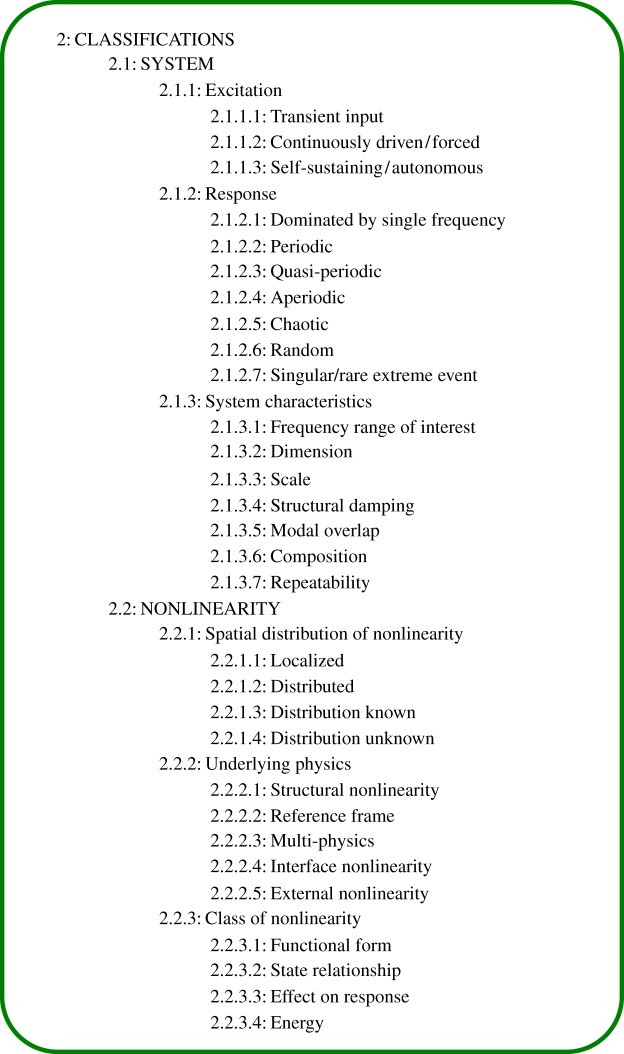
Outline of the *classifications* subheading structure, up to heading level four.

Classification is multi-dimensional, and there are many subheadings that could be used to identify the kind of system under investigation. An initial attempt has been made to list these subheadings; the broad structure is shown in figure 3. The first distinction is between *system*^(2.1)^ and *nonlinearity*^(2.2)^ classification. This is not to assume that there is an underlying linear system that can be identified, rather that some *classifications*^(2)^ are associated with the overall system (e.g. input *excitation*^(2.1.1)^ type) while others help to identify the underlying kind of nonlinearity (e.g. *spatial distribution of nonlinearity*^(2.2.1)^). The overall pattern of classifications associated with a given observed phenomenon defines its ‘signature’, which could potentially be used to find a good fit to a system *representation*^(3)^.

An initial difficulty to be noted is that academics often classify nonlinearities as either *weak*/*strong*^(2.2.3.3)^ or *local*/*distributed*^(2.2.1)^ (e.g. [26]), but once again these are terms that can be used to mean more than one thing. *Weak*/*strong* could refer to the degree of continuity of the *functional form*^(2.2.3.1)^ of the underlying nonlinear law, or the strength of the qualitative *effect on the overall system response*^(2.2.3.3)^; and *local*/*distributed* could refer to the *spatial distribution*^(2.2.1)^ of nonlinearity, or to localization in the space of linear modes or other degrees of freedom.

Vehicle *brake squeal* can occur as a *smooth growth*^(2.1.1.3.1)^ resulting from linear instability of the steady sliding solution, or it can be *triggered*^(2.1.1.3.2)^ by initial conditions or a large perturbation. Typically, the response is approximately *periodic*^(2.1.2.2)^ and *dominated by a single frequency*^(2.1.2.1)^, though observations of pairs of instabilities occurring simultaneously have sometimes been noted to give *quasi-periodic*^(2.1.2.3)^ behaviour [27]. The phenomenon spans the whole frequency range: *low-frequency*^(2.1.3.1.1)^ brake noise is often referred to as groan or judder; while *mid-*^(2.1.3.1.2)^ and *high-frequency*^(2.1.3.1.3)^ noise is usually classified as squeal [28]. Care is needed here as to how ‘low’ and ‘high’ are defined: while the definition could be in reference to human hearing, a more useful classification may be in terms of the modal series of an underlying linear system. In these terms, at *high* frequencies deterministic modal representations of structures are no longer appropriate and methods such as *statistical energy analysis*^(3.4.2.6)^ are much more useful for linear systems: recent extensions of this method for nonlinear systems are presented in this theme issue [7]. By this classification, *brake squeal* is therefore a *low*- or *mid-frequency* problem.

Although the actual *nuisance*^(4.1.4.3)^ arises from acoustic radiation, the underlying phenomenon is a *3D structural*^(2.1.3.2.2)^ problem where the structure consists of many *coupled subsystems*^(2.1.3.6.3)^. Typically, brake structures intrinsically have *low damping*^(2.1.3.4.1)^ and hence *low modal overlap*^(2.1.3.5.1)^, a concept that is closely related to the frequency classification above. One of the defining features of *brake squeal* is the *low repeatability*^(2.1.3.7.1)^ associated with the phenomenon: repeating an experiment under nominally identical conditions can yield qualitatively different behaviour.

The nonlinearity usually associated with *brake squeal* arises from *friction*^(3.1.1)^ *localized*^(2.2.1.1)^ to a *sub-region*^(2.2.1.1.3)^ of the disc (or in the case of some experimental studies, localized to a *single point*^(2.2.1.1.1)^ contact). Nevertheless, by far the most common approach to analysing the problem is to *linearize*^(3.2.2.3)^ the system and contact dynamics about the steady-sliding operating point and use the *eigenvalues*^(4.1.3.18)^ to predict *stability*^(4.1.3.19)^. If unstable, the subsequent growth in vibration is not always limited by *stick–slip*^(1.4.5.8)^ [29]: it can be that unilateral contact nonlinearity gives rise to *impacts/intermittent contact*^(2.2.2.4.1)^ (or near loss of contact) [24].

Some of the category headings reveal gaps in knowledge. For example, it is not clear to what extent *structural nonlinearity*^(2.2.2.1)^ plays a role (e.g. the brake pad *material*^(2.2.2.1.1)^ nonlinearities), and there are some questions as to whether *gyroscopic*^(2.2.2.2.2.1)^ effects sometimes play a significant role. The most conspicuous gap in knowledge concerns the *functional form*^(2.2.3.1)^ and *state relationship*^(2.2.3.2)^ of the frictional contact. For example, the range of friction laws that have been used in attempts to analyse *brake squeal* includes both *smooth*^(2.2.3.1.1)^, *history-dependent*^(2.2.3.2.2)^ laws (e.g. rate and state) and *non-smooth*^(2.2.3.1.2)^, *instantaneous*^(2.2.3.2.1)^ laws (e.g. Coulomb’s law): but this is an active area of research and is the subject of one of the papers in this theme issue [5], which tackles the question ‘What would be required to fill this gap in knowledge?’ Regardless of smoothness or memory effects of the underlying *functional form* of the constitutive law, it is clear that the overall effect of friction on the system behaviour is *strong*^(2.2.3.3.2)^, and that the presence of friction acts as an effective *energy source*^(2.2.3.4.3)^ translating energy from steady-sliding to high-frequency squeal noise.

### (c) ‘How can the system be represented?’

The general phenomenon of friction-induced vibration has been found to be extremely difficult to model: it is uncontroversial to say that no convincing predictive model of vehicle *brake squeal* currently exists. As mentioned, this is partly because *friction*^(3.1.1)^ itself is poorly understood and characterized. In terms of modelling, by far the most common approach is to carry out a *linearized*^(3.2.2.3)^ *stability*^(4.1.3.19)^ analysis of the steady-sliding operating point [30] (albeit with limited success). A variety of highly idealized models have been proposed that are claimed to ‘capture’ the phenomenon in some sense, and each gives some insight into the different mechanisms that can cause squeal. This itself is one of the modelling challenges: there are so many different mechanisms that can cause squeal that it is difficult to validate experimentally and isolate just one of these mechanisms. For example, it is known that a Coulomb *friction* model can induce *instability* in pairs of modes [31]; a velocity weakening model of *friction* can lead to a *linearized* negative damping effect [32]; disc rotation can cause *instability* through *gyroscopic effects*^(2.2.2.2.2.1)^ [33]; and uneven heat dissipation can lead to *thermal effects*^(2.2.2.3.2)^ such as thermoelastic instability [34].

*Time-domain simulation*^(3.2.2.1)^ is sometimes carried out to attempt to predict the fully developed nonlinear behaviour, and such models are often used to compare with more efficient schemes such as variations on the *harmonic balance method*^(3.2.2.7)^ (e.g. [35]). Intuitively, this makes sense: the system can be *linearized*^(3.2.2.3)^ about its operating point (steady sliding) in order to carry out *stability*^(4.1.3.19)^ predictions, but the fully nonlinear behaviour may include *non-smooth*^(2.2.3.1.2)^ and *strong*^(2.2.3.3.2)^ nonlinearity, so methods that rely on *perturbation*^(3.2.2.4)^ approaches (e.g. *normal forms*^(3.2.2.9)^ [36] or *Volterra series*^(3.2.2.10)^ [37]) are probably not applicable for predicting the nonlinear response.

The question of *uncertainty*^(3.4)^ representation is of particular interest and the effects of uncertainty have recently begun to receive attention (e.g. [38]). The system behaviour can be highly sensitive to these uncertainties, so this is an area of opportunity for research: to explore whether recent developments in, for example, *Bayesian system identification*^(3.4.2.2)^, *machine learning*^(3.4.2.3)^ or *information theory*^(3.4.2.7)^ can be used to give insight into the ‘twitchiness’ of *brake squeal*. Developments in the field of *Bayesian system identification* will be presented in one contribution to this theme issue [9], though not in relation to modelling squeal.

### (d) ‘What do you want to know?’

For a long time, the problem of squeal has been seen as an ‘urgent’ one requiring a ‘quick fix’ such as added damping or geometric adjustments. But there has been a growing realization that there is an underlying long-term research challenge that requires investment and collaboration even between competitors. The end-goal is ‘elimination’ but it is primarily a *nuisance*^(4.1.4.3)^ rather than being *safety critical*^(4.1.1.4)^, so models do not need to be overly conservative in their predictions, which may have a bearing on the choice of model. Of most interest to know is whether or not squeal occurs (e.g. *regime prediction*^(4.1.3.10)^ or *bifurcation thresholds*^(4.1.3.3)^). The details of how the *transient*^(4.1.3.16)^ waveform evolves during squeal initiation does not particularly matter in the way that it does for *bowed string instrument*^(1.3.1.1)^ dynamics. It is also apparent that there have not been many attempts to *actively control*^(4.2)^ squeal: cost, space constraints and the rotating reference frame of the disc each constrain significant intervention. However, some attempts have been made to use *dither*^(4.2.2.9)^ control (additive high-frequency signals applied to the brake structure) as a way of preventing squeal [39]. One mechanism by which this may be working is through *mean reduction in friction*^(1.4.6.12)^ [40].

## 5. Case study II: cable-stayed bridges

Having shown how the map can be usefully used in the context of one particular case study, let us show how different application domains lead to different answers when applying the map. To that end, we shall consider, somewhat more briefly, another example, where the industrial sector, the fundamental physics and the methods of analysis are all very different from the first example. Here the natural order to answer the fundamental questions, and hence the navigation route through the map, seems rather different too.

### (a) ‘What do you want to know?’

In the twentieth century *cable-stayed bridges*^(1.2.3.3)^ overtook suspension bridges as the method of choice for spanning all but the longest gaps between vertical supports, with the longest *cable-stayed bridge* spans now being over a kilometre in length. *Cable-stayed bridges* have a distinct advantage, as the force is distributed among many distinct inclined cables, rather than a single (pair of) cable(s) that has to be spun *in situ* as it is draped over the towers and for which anchorage at the two far ends of the span requires significant ground works in order to carry the forces associated with the entire bridge. Instead, the cables of *cable-stayed bridges* can be much less heavy duty and a number of different designs are possible, including fan, harp and side-spar configurations, that are adaptable to different site-specific and aesthetic considerations.

However, for the structural dynamicist in the *civil/built environment*^(1.1.10)^, *cable-stayed bridges* represent a significant challenge, because typically there are many tens if not hundreds of inclined cables, each of a different length. Each cable is subjected to a variety of forcing modalities, including *vortex shedding*^(1.4.5.3)^, combined wind–rain loading, as well as being globally coupled to each other through the motion of the deck, which itself receives vibration from multiple sources, not least the traffic passing over the bridge. Thus, at the purely linear level, the bridge can be thought of as an array of many coupled ‘tuning forks’, each with a different natural frequency. What is more, due to gravitational loading, inclined cables are known to exhibit significant *geometric nonlinearity*^(2.2.2.1.4)^.

Fundamentally, then, the sorts of question the bridge designer might ask are: ‘Will the design of the bridge lead to inherent oscillatory *instabilities*^(4.1.3.19)^?’ ‘Will any such oscillations be *safety critical*^(4.1.1.4)^?’ ‘Will they lead to *fatigue*^(4.1.1.1)^?’ ‘Will they just be a *nuisance*^(4.1.2)^ that will cause potential passengers to lose confidence in using the bridge?’ In many parts of the world, one also has to consider the *response of the structure to earthquakes*^(1.2.1.8)^. A bridge owner or operator might ask in extreme conditions such as storm winds or a large group of pedestrians using the bridge: ‘Is the bridge safe to operate; should I close it?’

### (b) ‘How can the system be represented?’

An even more practical question at the design stage might be: ‘How faithful do I need to make my computer model of the bridge in order to correctly capture such potential *instabilities*^(4.1.3.19)^?’ For example, many finite-element models of *cable-stayed bridges* used in practice describe each cable as a single linear element. Is this sufficient? However, the nonlinear behaviour of even a single inclined cable is a research topic in itself (see, for example, the contribution by Neild *et al.* [8]). A full formulation involves a partial differential equation model that accounts for the *geometric nonlinearity*^(2.2.2.1.4)^ due to sag. These nonlinear terms not only provide coupling between different planar modes of vibration but also allow for coupling between in-plane and out-of-plane modes. Such coupling can lead to circular motion, often described as *galloping, whirling or ballooning*^(1.2.2.6)^.

Another problem is how to represent *damping*^(3.1.4)^ and the interface between bridge and deck [41]. The kind of mounting used in practice typically involves some *frictional damping*^(1.4.2.2)^, and the cables themselves have internal damping that is a *side-effect of the assembly*^(1.4.2.2.2)^ due to individual strands rubbing against each other. Then there is the question of the *fluid–structure interaction*^(2.2.2.3.1)^ of such cables exposed to wind. There are documented excitation mechanisms due to *vortex shedding*^(1.4.5.3)^, which has been hypothesized to be exacerbated by the effective bluff-body profile formed by rivulets of water descending the cable during wind–rain loading. *Fluid–structure interaction*^(3.1.5)^ is also well known to have *fluid-damping*^(1.4.2.1)^ effects. Then there are issues to do with how to represent the motion of and loading on the deck itself. For example, the undesired horizontal vibrations of London’s Millennium Bridge has led to new understandings of the possible effects of pedestrian loading, and its possible link to the *synchronization*^(1.4.5.5)^ behaviour of crowds, but a simplified model of how to represent such loads is not yet clear.

### (c) ‘What do you observe?’

Even models of inclined cables attached to a deck undergoing pure sinusoidal motion can show complex nonlinear behaviour. One of the particular computations is that, because of the inclined nature of the cable’s axis, both *parametric resonance*^(1.4.6.7)^ and *direct resonance*^(1.4.6.5)^ can occur [42]. In addition, for a taught cable, the effect of gravity is typically a *weak perturbation*^(2.2.3.3.1)^ of the symmetric situation, and so in-plane and out-of-plane modes are usually only mildly detuned. Furthermore, the nature of the spectrum of a cable is such that the second mode of vibration is typically close to twice the frequency of the first mode, leading to an *internal resonance*^(1.4.6.6)^ and an interaction between *parametric* (twice frequency) and *direct* (harmonic) mechanisms of *instability*^(4.1.3.19)^ [43–45]. So the system naturally has multiple modes that are in near-resonance, and so complex sequences of *mode locking*^(1.4.1.3)^ and *mode jumping*^(1.4.4.3)^ can be observed in addition to *hysteresis*^(1.4.6.1)^, where a different response is seen under increase in excitation frequency than under decrease. For sufficiently large-amplitude excitation, *chaotic*^(1.4.6.10)^ responses can be seen.

These kinds of dynamics are often studied using reduced models that exploit either the *method of multiple scales*^(3.2.2.5)^, *harmonic balance*^(3.2.2.7)^ or *normal form*^(3.2.2.9)^ analysis [46,47].

### (d) ‘What do you know?’

Unlike other engineering structures (such as, for example, helicopters, where rotor frequencies are deliberately designed to avoid frequencies associated with fuselage modes), resonances cannot be avoided in cable-stayed bridges, because there are cables of so many different lengths. The trick instead is to make sure that any undesirable vibration mode, of either deck or cable, is suitably damped. For the practical bridge designer, *damping*^(2.1.3.4)^ is generally one’s friend. Oscillations of the Millennium Bridge were alleviated by the retrofitting of dampers. Tests on the Second Severn Crossing during construction resulted in additional ties being added to the cables to avoid unwanted *large-amplitude*^(2.2.2.1.3)^ oscillations [48]. A lot has been learned since the well-documented failure of the Tacoma Narrows bridge in the 1940s (itself a suspension bridge, not a *cable-stayed bridge*).

## 6. Mapping the theme issue contributions

Having presented some illustrative details of the map contents, it is interesting to look in more detail at some of the patterns that emerge from the theme issue contributions shown in figure 2.

One immediately notable feature is a pair of conspicuous gaps common to most papers towards the end of 2.2.2 and 4.2.1/2 (at about eight o’clock and one o’clock, respectively, in figure 2). The gap at 2.2.2.5 corresponds to systems with an external source of nonlinearity, for example, arising from *active control*, *actuators*, *electromagnetics* or *switches*. The gap at 4.2 flags a related issue, since it corresponds to various *control methods*. One paper in this theme issue touches briefly on the issue of control [6] within the contexts of vibration isolation and of dynamic range compression in the cochlea, but there are many other important applications and control techniques that are not included, for example, *sliding mode control*, *H-infinity control* or *model predictive control*. This lack of focus on ‘advanced’ control techniques reflects some commonality between contributors, and at face value is simply a gap in the subject coverage, but another possible interpretation is that such control systems are not common for suppression of nonlinear vibration. Possibly, this highlights an area that has potential for further investigation, but it could also be a warning that such methods may not be cost effective, or are simply too difficult, to develop in the typical contexts where nonlinear structural vibration occurs. For example, it has been shown that local collocated proof-mass systems, such as *skyhook dampers*^(4.2.2.1)^, rival the performance of advanced distributed systems but with the added advantage of simplicity [49].

It can be noted that some of the rings within figure 2 are densely populated while others are more sparse. The contribution with greatest coverage corresponds to the eighth ring [9]. This paper concerns system identification, with an emphasis on Bayesian methods: finding models and their parameter values to represent a given phenomenon, with their associated uncertainty distributions. The proponents of this approach contend that it can be applied to nearly everything within the field of nonlinear structural dynamics (and indeed any domain where models are developed in conjunction with field data). This connection to ‘nearly everything’ is appealing, and there is certainly scope for wider application of the methods that are under development. There are two significant impediments to widespread adoption of these methods: a psychological barrier, as the language of Bayesian inference is not generally well known in industry; and also the fact that such methods still require efficient underlying numerical models that can feasibly be computed many times. The developments presented in this theme issue [9] go some way to minimizing the number of simulation runs necessary.

**Figure 4.**
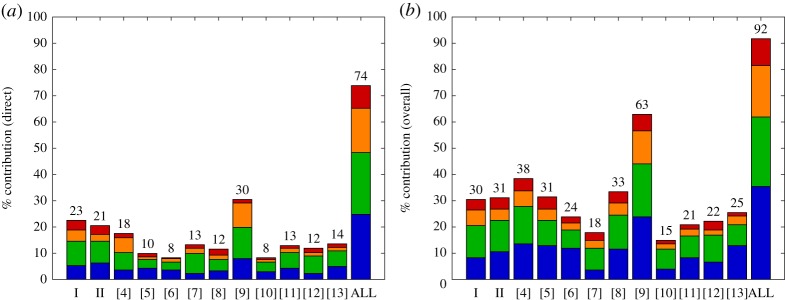
Breakdown of percentage contribution of each paper to the overall map: (*a*) directly related and (*b*) either directly or indirectly related. The colours correspond to the four main groups: *observations* (blue), *classifications* (green), *representations* (orange) and *outcomes* (red). The two case studies are labelled as ‘I’ (vehicle brake squeal) and ‘II’ (cable-stayed bridges), and the contributions to this theme issue are labelled by their reference number within this introductory article.

Other rings appear rather sparse, such as rings 9 and 11 [10,12]. This is partly an artefact of fragmentation, and figure 4 shows the actual percentage contribution of each article, colour-coded by group heading. This shows that each paper relates directly to about 10–20% of the map (exception [9] aside), and indirectly to 20–30%.

## 7. Conclusion

This article has documented an attempt to create a map, or ‘consultant’s checklist’, associated with the field of nonlinear structural dynamics. The idea is that the map should be a living document, and this article should just be viewed as presenting a current snapshot. The map was inspired by a collaboration between five UK universities, eight industrial collaborators and a number of international advisors, where it became apparent that different experts used specific terminology and jargon according to their particular areas of specialism. This led to a collaborative effort to put these terms to paper and assemble them into some form of structure. The result is a hierarchical list of labels that summarize many of the key concepts within the field. It is not a finished product and the authors actively invite readers to re-use and add to the map, which can be found in various formats in the electronic supplementary material. Accompanying the map is a list of questions that help to identify key features of a phenomenon and challenge prior assumptions.

In brief, the map provides a structured way of: (a) highlighting the features of the phenomenon that are commonly *observed* (e.g. *self-sustained vibration*^(1.4.5.2)^); (b) *classifying* the problem using higher-level abstractions (e.g. *spatially localized nonlinearity*^(2.2.1.1)^); (c) identifying common system *representations* and potential areas for new research (e.g. *uncertainty quantification*^(3.4)^); and (d) defining what *outcomes* are actually of most interest for a particular application (e.g. *bifurcation thresholds*^(4.1.3.3)^). This process illustrates how the map can be used to identify interrelationships between subjects in a systematic way, potentially leading to identification of suitable methods for approaching the problem, and of identifying relevant experts (perhaps by revealing areas that are important but for which in-house knowledge is limited).

The map and the list of questions have been illustrated in the context of two specific applications: *vehicle brake squeal*^(1.2.2.1)^ and the dynamics of *cable-stayed bridges*^(1.2.3.3)^. These examples are indicative of how the map could be used to structure a set of research and deployment questions. This could enable researchers, developers or users to identify possible ways of addressing their problems, thereby seeking a number of feasible pathways that could lead to some desired outcomes. Conversely, the examples show how aspects of an application problem could be interrogated and classified, to provide information on how a given issue was addressed. Potentially, the map could be used to represent some aspects of why the chosen solution methods were used. The associations within each category could then provide information on possible alternative approaches that could also have been used.

The map has also been used to give an introductory overview of the other articles in this theme issue. Each contributing author has identified their topic’s connections to the map. Together, the articles span a significant segment of the field of ‘nonlinear structural dynamics’, and the map draws out some interesting themes.

We trust that in reading the rest of the contents of this theme issue the reader might be inspired to think of their own area of interest related to nonlinear structural dynamics and to attempt to use the map to classify it. In doing so, you will no doubt find some terms or areas of classification missing from the map’s index, as has invariably been the case with each user so far. One key development might be to define different types of associations that relate concepts, and use these to link concepts within and between categories, transforming the structured list of labels into a network of concepts. We would therefore encourage you to get in touch, using the email address of the first author, so that we can update the map, and you can participate in what we hope will remain a worthwhile, ongoing research-community-led exercise. In a sense, we hope that this will help pioneer a new ‘crowd-sourced’ collaboration methodology, within an important part of engineering science.

## Supplementary Material

Online supplementary material
