# Supplementary material for: The landscape of nonlinear structural dynamics: an introduction
Source: Philos Trans A Math Phys Eng Sci. 2015 Sep 28;373(2051):20140400. doi: 10.1098/rsta.2014.0400 (PMC4549938; doi:10.1098/rsta.2014.0400)
Supplement: Online supplementary material [file rsta20140400supp1.pdf]

Online supplementary material

The following files can be found in the online supplementary material (files with extension *.vdk can be opened using designVue [1]):

- `map_overview_designVue (*.vdk / *.pdf)`: a summary of the overall structure of the map, which was used to generate Figure 1;
- `fig_author_contibutions_with_labels.png`: a high resolution image of Figure 2 including all map labels and reference numbers;
- `map_full_designVue (*.vdk / *.pdf)`: a current snapshot of the full map;
- `map_case_studies_designVue (*.vdk / *..pdf)`: Map of the two case studies. The links in the *.vdk file can be explored interactively using designVue. The *.pdf file is a zoomable screenshot from designVue;
- `map_full (*.xlsx / *.pdf)`: a current snapshot of the full map as a list with reference numbers;
- `map_C1_vehicle_brake_squeal.pdf)`: Map of Case Study I: Vehicle brake squeal. A list of labels connected to *vehicle brake squeal*, with reference numbers;
- `map_C2_cable-stayed_bridges.pdf)`: Map of Case Study II: Cable-stayed bridges. A list of labels connected to *cable-stayed bridges*, with reference numbers;
- `map_author_contributions_data (*.xlsx, *.pdf)`: Raw data of author contributions to this Theme Issue, with reference numbers;
